# Biomimetic Silica Encapsulation of Lipid Nanodiscs and β-Sheet-Stabilized Diacylglycerol Kinase

**DOI:** 10.1021/acs.bioconjchem.1c00260

**Published:** 2021-07-21

**Authors:** Friedrich Bialas, Christian F. W. Becker

**Affiliations:** Institute of Biological Chemistry, Faculty of Chemistry, University of Vienna, Währinger Straße 38, 1090 Vienna, Austria

## Abstract

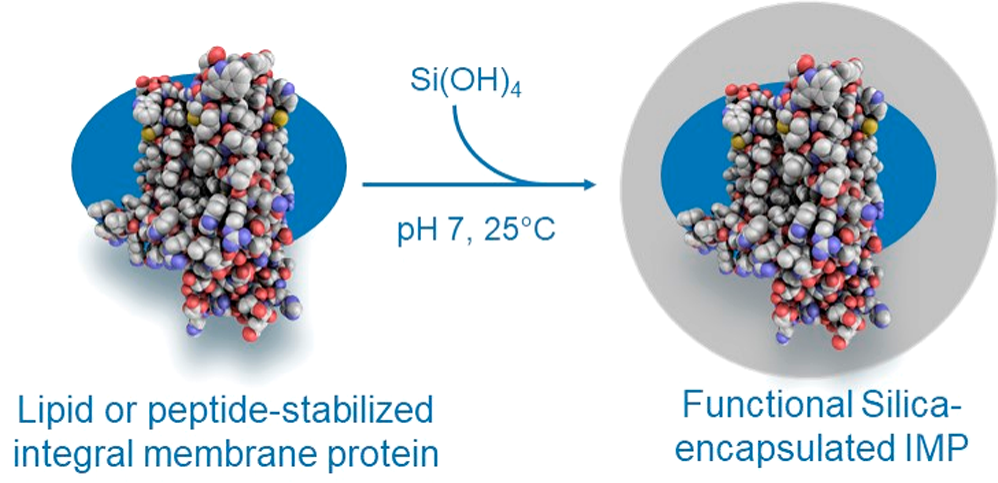

Integral membrane proteins (IMPs) comprise highly important classes of proteins such as transporters, sensors, and channels, but their investigation and biotechnological application are complicated by the difficulty to stabilize them in solution. We set out to develop a biomimetic procedure to encapsulate functional integral membrane proteins in silica to facilitate their handling under otherwise detrimental conditions and thereby extend their applicability. To this end, we designed and expressed new fusion constructs of the membrane scaffold protein MSP with silica-precipitating peptides based on the R5 sequence from the diatom *Cylindrotheca fusiformis*. Transmission electron microscopy (TEM) and atomic force microscopy (AFM) revealed that membrane lipid nanodiscs surrounded by our MSP variants fused to an R5 peptide, so-called nanodiscs, were formed. Exposing them to silicic acid led to silica-encapsulated nanodiscs, a new material for stabilizing membrane structures and a first step toward incorporating membrane proteins in such structures. In an alternative approach, four fusion constructs based on the amphiphilic β-sheet peptide BP-1 and the R5 peptide were generated and successfully employed toward silica encapsulation of functional diacylglycerol kinase (DGK). Silica-encapsulated DGK was significantly more stable against protease exposure and incubation with simulated gastric fluid (SGF) and intestinal fluid (SIF).

## Introduction

Biological membranes are highly organized and functional structures separating regions of varying metabolic activity.^1^ Integral membrane proteins (IMPs) equip membranes with various crucial functions including passive and active transport, signal transduction, and more, but their investigation is complicated by the difficulty to stabilize them in solution.^2^ Several strategies have been devised to facilitate IMP handling in the lab and beyond for biotechnological as well as biomedical applications.^3,4^

Conventional methods to increase the expression yield of membrane proteins and to improve their solubility are based on genetic modification of the protein itself. Either a soluble protein is fused to the protein of interest, or mutations are introduced to overall stabilize the membrane protein against the loss of structure and, when changing surface properties as well, to increase solubility. However, such modifications can impede function.^5^ Consequently, methods for membrane protein stabilization without genetic modification have been developed,^6,7^ including the use of lipid-bilayer nanodiscs^8^ and of peptide based systems such as beltides,^9^ peptidiscs,^10^ or the amphiphilic BP-1 peptide.^10−12^

**Figure 1:**
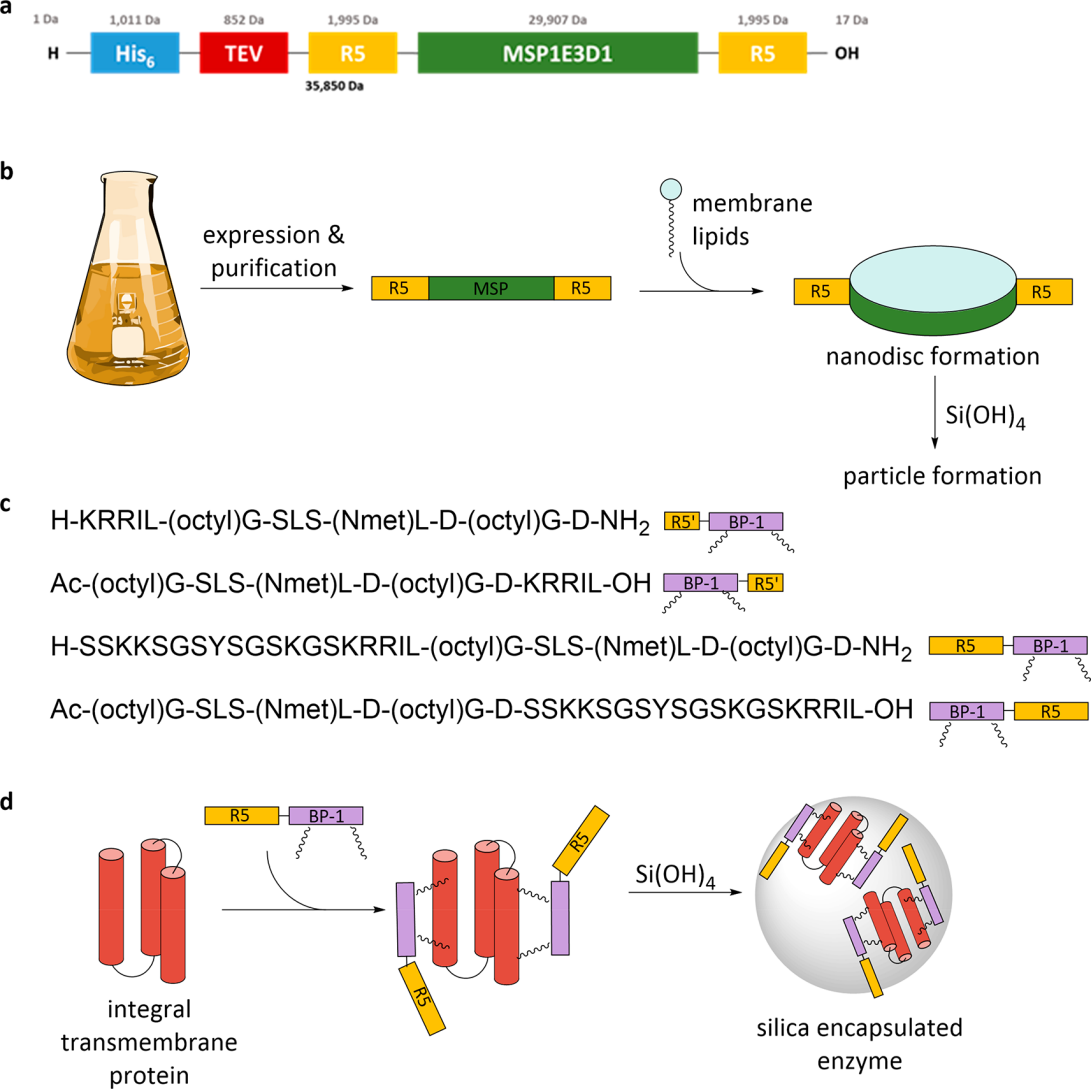
Schematic representation of the modified membrane scaffold protein sequence comprising a removable purification tag (His_6_ + TEV), two R5 peptides, and the MSP (a); process of generating silica-stabilized nanodiscs (b); sequences of the BP-1 peptides fused to different R5 peptides (R5′ corresponds to KRRIL) KRRIL-BP-1, BP-1-KRRIL, R5-BP-1, and BP-1-R5 (c); and membrane protein encapsulation in silica using BP-1-R5 constructs (d). New variants of the R5-membrane scaffold protein (MSP) fusion protein, shown in yellow and green, are used to create nanodiscs. Modified BP-1 peptides, shown in purple, can directly be used for the encapsulation of membrane proteins in silica.

Nanodiscs are protein-stabilized lipid bicelles that mimic their natural environment and are successfully used in the purification,^13^ stabilization, and structure determination of membrane proteins.^8^ They are created from the amphiphilic membrane scaffold protein (MSP) and phospholipids.^14^ In order to derive a generic approach for the encapsulation of IMPs into silica-stabilized membrane nanodiscs, we have designed and expressed new variants of MSP fused to a silica-precipitating peptide based on the R5 silaffin peptide from *Cylindrotheca fusiformis* (Figure 1a,b).^15^

To enable the reconstitution of IMPs and their subsequent encapsulation in silica particles in the absence of lipids, a modified BP-1 peptide was developed as well. Two different silica-precipitating tags were fused to the BP-1 peptide: the full-length R5 peptide from the diatom *C. fusiformis* and a truncated version based on the C-terminal amino acids KRRIL only (R5′, Figure 1c). Approaches based on silaffin peptide directed biomimetic silica precipitation have been previously used to encapsulate biological structures such as eukaryotic cells and biomaterials^16^ as well as for the generation of protected enzymes and microcompartments^17,18^ since their discovery more than 20 years ago.^19,20^ Genetic manipulation to generate diatoms with new enzymatic functions based on their silica deposition machinery has also been reported^21^ as well as the use of synthetic silaffins to encapsulate soluble proteins.^15,22^ Here, we aim for the encapsulation of a functional membrane protein in a fully controlled lipid or detergent environment without direct modification of the protein to allow transfer of this approach to other membrane proteins.

The integral membrane enzyme diacylglycerol kinase (DGK) was used as a model system to create silica particles containing a functional integral membrane enzyme that can generate phosphatidic acid from diacylglycerol substrate (Figure 1d). *Escherichia coli* DGK comprises 121 amino acids and forms a homotrimer in its native membrane environment.^23^ It can be purified from *E. coli* and is also accessible by chemical protein synthesis.^24^ The enzyme is an unusual kinase and transfers the γ-phosphate of adenosine triphosphate (ATP) to diacylglycerols carrying variable fatty acids thereby producing the corresponding phosphatidic acid and adenosine diphosphate (ADP).^25^ The active site is a composite or shared active site, which consists of residues contributed by different monomers, therefore requiring the complete assembly of the trimer to become functional.^26^ The enzyme has been extensively used as a model for membrane protein folding and can insert into preformed lipid vesicles.^27^ It is also active when reconstituted in nanodiscs but not in micelles.^28^ Here, we demonstrate the encapsulation of the active integral membrane protein DGK in biomimetic silica with the aim to extend the use of functionally encapsulated IMPs to applications as catalysts in biotechnology or as sensors in biomedical applications, similar to the scope of applications for encapsulated soluble enzymes.^29,30^

## Results and Discussion

### R5-MSP-R5 Fusion Construct Enables the Formation of Silica-Encapsulated Nanodiscs

The silica-precipitating R5 peptide was genetically fused to both the N- and the C-terminus of the membrane scaffold protein (MSP, variant MSP1E3D1),^31^ which stabilizes the lipid-bilayer nanodiscs in solution. For ease of purification, the insets were designed to introduce a TEV-protease cleavage site (ENLYFQG) in between the N-terminal His-tag and the desired R5-MSP-R5 protein sequence (Figure 1a; for details about cloning, sequencing, and expression, please see the Supporting Information, Figures S1 and S2).

**Figure 2:**
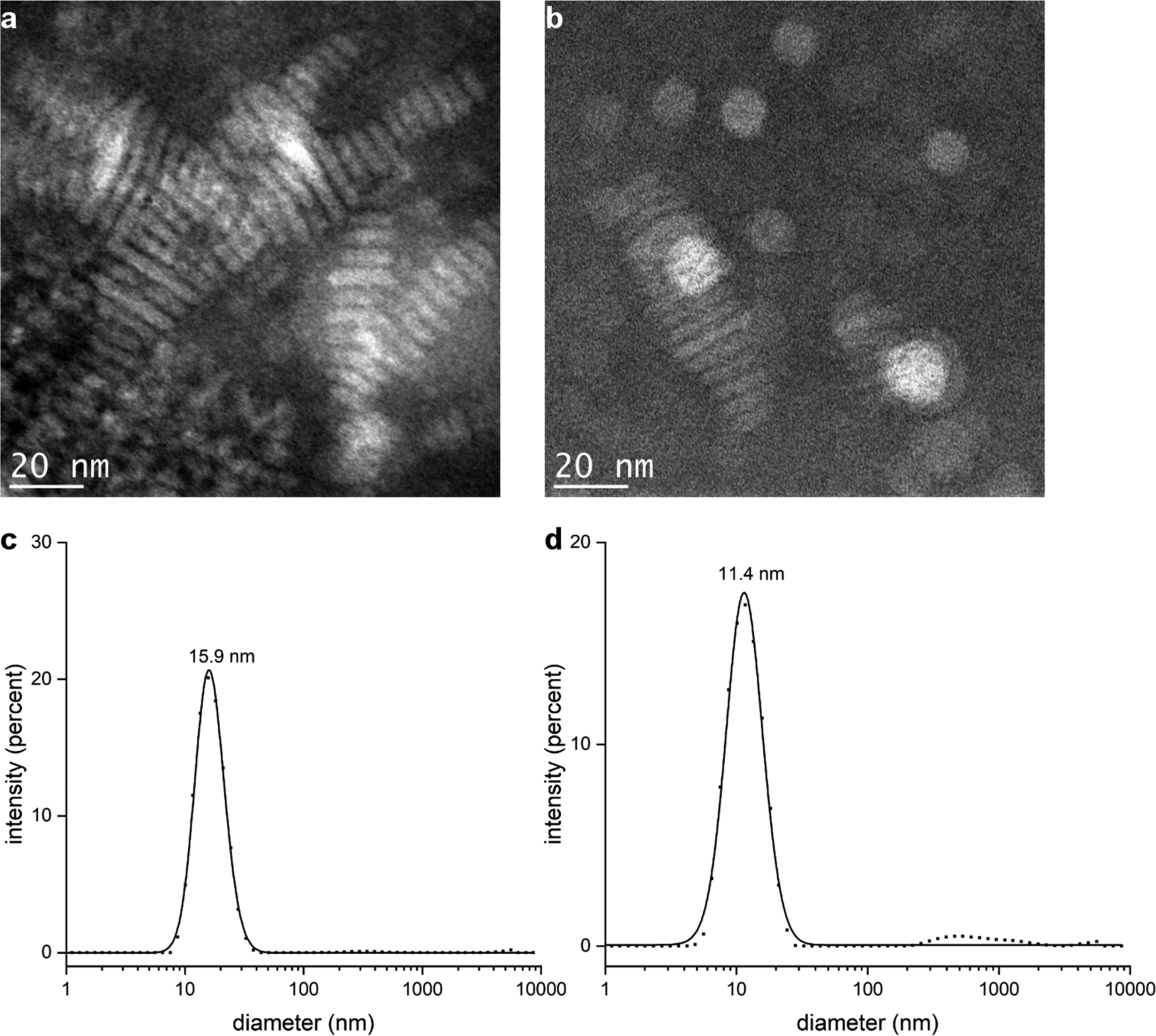
Transmission electron micrographs of nanodiscs created with the two MSP variants. (a) MSP nanodiscs, which are visible mainly as toppled stacks. (b) R5-MSP-R5 nanodiscs, which are a mixture of nanodisc stacks and individual nanodiscs lying flat. DLS size distributions of MSP nanodiscs (c) and R5-MSP-R5 nanodiscs (d) prepared with DOPC.

The R5-MSP-R5 construct was successfully expressed in *E. coli* BL21 (DE3) Rosetta 2 and purified by Ni-NTA affinity chromatography (Figure S3). Treatment with TEV protease and an additional Ni-NTA purification step provided the desired R5-MSP-R5 protein with the expected molar mass of 33 972 Da (Figure S3). In a typical expression, 8.5 mg/L of protein was obtained of which 6.1 mg/L (76%) was recovered after TEV protease cleavage. The production of unmodified MSP for a direct comparison was performed according to Bayburt et al.^14^ CD measurements of the two MSP variants indicate an identical α-helical conformation (Figure S4). Thus, the presence of the R5 tags does not seem to influence the native conformation of the MSP protein, indicating that the formation of nanodiscs should be possible with this fusion protein. Nanodiscs were prepared using 1,2-dioleoyl-*sn*-glycero-3-phosphocholine (DOPC) as a membrane lipid and sodium cholate as a detergent. Transmission electron microscopy confirmed the formation of nanodiscs with both MSP variants, which appear either as circles, when the nanodiscs are lying flat on the carbon film surface, or as alternating dark and light stripes, indicating toppled stacks of nanodiscs (also described as “rouleaux”,^32^Figure 2a,b). The nanodiscs made with the MSP protein have an average diameter of 14.1 ± 0.2 nm (average of 70 individual discs), whereas those made from the modified R5-MSP-R5 are slightly larger with 14.8 ± 0.3 nm (average of 92 individual discs). Solutions of the nanodisc variants were subjected to dynamic light scattering (DLS) to measure their hydrodynamic diameters. For the MSP nanodiscs, a maximum at 15.9 nm was found (Figure 2c), and for R5-MSP-R5, one at 11.4 nm was found (Figure 2d). The discrepancy in nanodisc diameters between measurements in solution and TEM is most likely based on the increased polarity of the R5-MSP-R5. The highly charged R5 peptides should be solvent exposed at the outer layer of disc structures (Figure 1) and lead to a thinner solvent layer. These results indicate that individual nanodiscs are present in solution.

To further confirm the formation of nanodiscs with R5-MSP-R5, atomic force microscopy (AFM) images on a mica surface were taken, and nanodiscs appear as dots in the resulting pictures (Figure S5a). A line plot of the AFM images reveals the thickness of the discs, which is approximately 6 nm and thus in good agreement with the values published for comparable MSP nanodiscs (Figure S5b).^14^

To investigate the silica precipitation of R5-MSP-R5 alone and of nanodiscs formed with R5-MSP-R5, samples were transferred into 50 mM potassium phosphate buffer at pH 7 at a concentration of 1 mg/mL. The addition of freshly hydrolyzed silicic acid (originating from TMOS, hydrolyzed for 4 min) initiated silica formation. After incubation at room temperature for 30 min, TEM samples were collected. Under these conditions, silica particles with both R5-MSP-R5 protein and R5-MSP-R5 nanodiscs were obtained. MSP protein and MSP nanodiscs not containing the R5 peptide did not induce silica precipitation.

**Figure 3:**
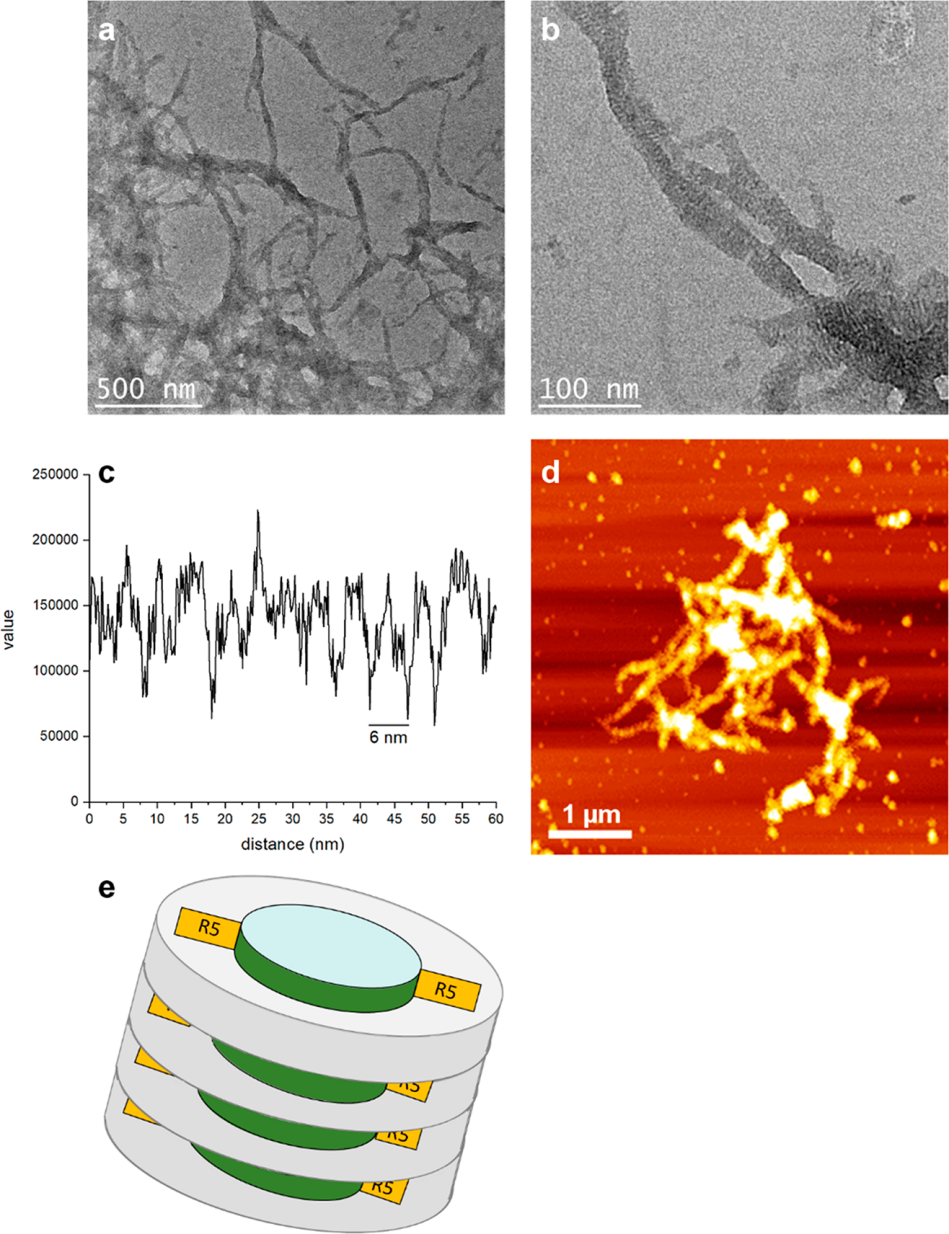
(a, b) Transmission electron micrographs of silica particles precipitated with the R5-MSP-R5 nanodiscs at increasing magnification. (c) A line plot of brightness values along one fibril shows that striations are approximately 6 nm apart. (d) Atomic force microscopy image of silica-covered nanodisc fibrils (d). (e) Illustration of stacks of nanodiscs encapsulated by a silica shell as seen in TEM pictures in panel b.

Silica precipitates formed with R5-MSP-R5 alone are amorphous and do not give rise to defined larger structures (Figure S6). In contrast, silica-encapsulated R5-MSP-R5 nanodiscs lead to a fibrillar morphology as visualized by TEM analysis of the precipitate. They possess a spongelike structure composed of striated fibers that correspond to stacks of nanodiscs covered with silica (Figure 3a,b,e). Measurements of 16 individual fibers give an average diameter of 23.8 ± 1.1 nm, sufficient for the accommodation of R5-MSP-R5 nanodiscs with a diameter of 14.8 nm. A line plot shows that the striations seen on the fibrils are approximately 6 nm apart (Figure 3c), further indicating that nanodisc stacks form the core of the fiber (Figure 3e). This sample was also imaged using AFM confirming the fibrillary structure of the silica-covered nanodiscs but less finely resolved (Figure 3d).

After succeeding to generate silica-covered lipid nanodiscs, we continued with the incorporation of DGK as a model IMP into these structures. To this end, DGK was expressed in *E. coli* and purified according to the protocol used by Lau et al.^25^ However, all attempts to incorporate functional DGK into MSP or R5-MSP-R5 nanodiscs failed. Incorporation was assessed based on affinity purification of nanodiscs that should contain DGK with a His_6_ tag (for details, see the SI, Figure S8). To the best of our knowledge, the incorporation of DGK into lipid nanodiscs has not yet been reported. This might be due to the specific properties of DGK and its strong dependence on bilayer thickness^33^ and the properties of lipids (lipid mixtures) used in general.^34^ Future attempts to achieve this goal will include different lipids and/or variants of MSPs.^35^ However, as this approach requires customized solutions rather than being generally applicable to IMPs, we switched to an alternative strategy using the previously described BP-1 peptides to stabilize IMPs in the absence of lipids. To this end, we modified the BP-1 peptide with silica-precipitating peptides based on R5 to obtain functional DGK embedded in silica.

### R5-Modified BP-1 Peptides Enable the Encapsulation of Functional DGK in Silica Particles

Four new constructs based on the BP-1 peptide linked to two different silica-precipitating tags were designed. The full-length R5 peptide from *C. fusiformis* or its five N-terminal amino acids (R5′) were attached at either the N- or the C-terminus of BP-1 (Figure 1c,d).^11^ All four constructs and the unmodified BP-1 peptide were successfully synthesized by solid-phase peptide synthesis (SPPS; as previously described by Tao et al.^11^) and purified by RP-HPLC. All peptides were obtained in isolated yields of 5–88% and in high purity (Figure S9 and Table S2). Lower yields were caused either by the occurrence of major deletion products during synthesis or by the low solubility of the constructs, which prevented efficient elution during HPLC purification.

All BP-1 constructs were analyzed by CD spectroscopy, and unmodified BP-1 showed a minimum at the 220 nm wavelength in its spectrum (Figure S10) indicating a predominant β-sheet conformation,^36^ as reported before.^11^ The R5-BP-1 hybrid constructs give similarly shaped CD curves clearly demonstrating that the properties of BP-1 control secondary structure formation. However, the C-terminal addition of R5 to BP-1 in BP-1-R5 gives rise to an additional minimum between 200 and 210 nm, which might be due to self-assembly induced by the C-terminal RRIL-motif. Accordingly, the BP-1-R5′ peptide shows a similar CD trace with an even less pronounced minimum at 220 nm (Figure S10b).

**Figure 4:**
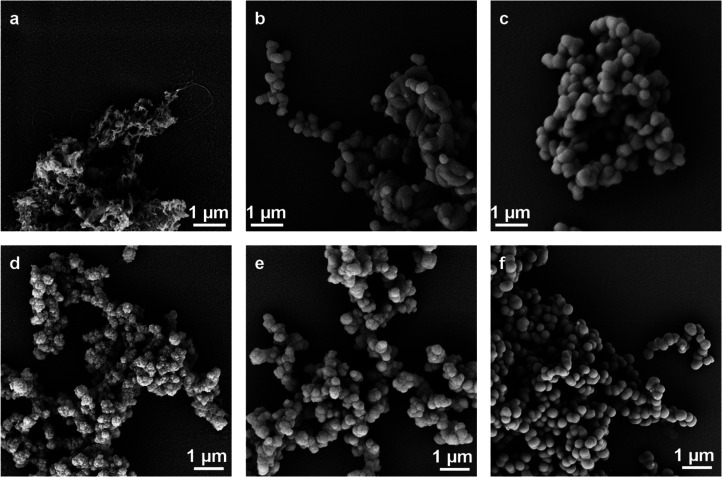
Silica particle formation with modified BP-1 peptides. Scanning electron micrographs (10 000× magnification) of silica particles precipitated with the peptides R5′-BP-1 (a), R5-BP-1 (b), and BP-1-R5 (c) and silica particles precipitated with the DGK protein stabilized with R5′-BP-1 (d), R5-BP-1 (e), and BP-1-R5 (f).

All BP-1 variants were tested toward their ability to induce silica precipitation from silicic acid under biomimetic conditions (for details, see the SI). As expected, no silica precipitation was observed for the unmodified BP-1 peptide. The BP-1-R5′ peptide proved insoluble under the testing conditions and therefore could not be used for silica precipitation. In contrast, silica precipitates were observed for the R5′-BP-1, R5-BP-1, and BP-1-R5 variants, and they were characterized by scanning electron microscopy (SEM, Figure 4a–c). The different morphologies of the precipitates are revealed under the microscope: fibrillar silica material was obtained with R5′-BP-1 (Figure 4a), which is in agreement with the β-sheet forming properties of BP-1. In contrast, R5-BP-1 led to spherical particles (Figure 4b). The average diameter of the single nanospheres was 316 ± 8 nm as determined by measurements of 46 individual particles. BP-1-R5 generated almost perfectly shaped spherical silica particles (Figure 4c), which were slightly larger than the ones obtained with R5-BP-1. Measurements of 36 individual particles showed an average diameter of 408 ± 8 nm. These different morphologies can be explained by the size of the silaffin-based peptides attached to BP-1. R5, comprising 19 amino acids, seems to control the assembly properties of the hybrid R5-BP-1 and BP-1-R5 peptides (with BP-1 consisting of only eight amino acids). The much shorter RRIL motif in R5′-BP1 is forced into fibrillar structures by the BP-1 driven assembly process on the other hand. For all constructs, the accessibility of the RRIL motif is important due to its critical role in silica precipitation.^37−39^ Burying the RRIL motif in the middle of the R5-BP-1 construct could explain the less homogeneous particles generated with this hybrid peptide, whereas the more exposed RRIL motif in BP-1-R5 could lead to a more symmetrical peptide assembly and thus a more uniform particle shape. We also analyzed how many of the peptides were incorporated into the silica particle by analyzing the supernatant of precipitation reactions on HPLC (Figure S11). For R5-BP-1 and BP-1-R5, quantitative incorporation into silica was observed whereas, for R5′-BP-1, incorporation could not be monitored due to solubility issues even though silica precipitation was still possible (Figure S11).

The reduced solubility of both R5′ hybrids with BP-1 compared to the larger R5-BP-1 hybrids is most likely caused by the fact that the short KRRIL (R5′) peptide cannot fully solubilize the hydrophobic lipid-containing BP-1 peptide. Here, we decided to proceed with incorporating DGK into the R5-BP-1 and BP-1-R5 derived silica particles, in which the much longer R5 peptide (19 aa) overcomes the solubility issues of BP-1, by initially generating DGK stabilized by our modified BP-1 peptides. To achieve this goal, a solution of DGK was diluted to 1 mg/mL concentration in DGK dialysis buffer at a total volume of 200 μL. 20 equiv of the respective BP-1 peptide variant were added, and the mixture was incubated for at least 30 min. Dialysis against 200 mL of phosphate buffer (50 mM sodium phosphate, 300 mM NaCl, pH 8 at 4 °C with two buffer exchanges) led to the removal of detergent and stabilization of DGK with R5-BP-1 variants. Silica particles were subsequently prepared by adding 10 μL of freshly prepared silicic acid (960 μL of 1 mM HCl was mixed with 40 μL of TMOS and incubated for 4 min) to 90 μL of the dialyzed solution and by incubating the mixture at room temperature for 30 min. After centrifuging the precipitation mixture for 5 min at 14 000 rpm in a table-top centrifuge and removing the supernatant, the isolated particles were washed with 100 μL of water before further use.

As expected, the DGK complexed by unmodified BP-1 did not lead to the formation of silica particles, but all other variants did. Again, scanning electron microscopy revealed that the particles were of different morphologies, depending on the hybrid construct used. The particles containing DGK and R5′-BP-1 were irregularly structured, most likely again related to solubility issues of the R5′-BP-1 peptide. These particles did not allow the determination of single particle dimensions from micrographs constituting another reason to discontinue using this hybrid peptide (Figure 4d). Interestingly, no fibrillar structures were visible anymore indicating that the long-range order usually induced by the BP-1 peptide in this peptide was disrupted by interactions with DGK, similar to results previously reported for the IMP stabilization of this peptide.^11^ Particles containing DGK and R5-BP-1 were more homogeneous and spherical with an average diameter of 345 ± 8 nm (Figure 4e). DGK containing BP-1-R5 particles were spherical and on average 697 ± 6 nm in diameter (Figure 4f). EDX spectra clearly indicated the presence of silicon in all samples after silica precipitation confirming that all structures analyzed here contain silica (Figures S13–S15).

The encapsulation efficiency using R5-BP-1 and BP-1-R5 was determined by the quantitative SDS-PAGE evaluation of samples of the starting DGK solution and the supernatant after precipitation (Figure S12). To evaluate the effects of individual components, we included R5 and BP-1 peptides alone as controls. The intensity decrease of the protein bands in the supernatant of silica precipitation reactions corresponds to the fraction entrapped in silica particles. Band intensities from three separate precipitation reactions were analyzed and gave the encapsulation efficiencies listed in Table 1. The BP-1-R5 peptide clearly gave the highest DGK encapsulation efficiency and the least batch-to-batch variations. Based on these parameters, we selected BP-1-R5-stabilized DGK for all further assays. BP-1 alone did not lead to significant silica precipitation, and in turn, only a small reduction of DGK in the supernatant was observed. As expected, R5 peptide alone led to silica precipitation. Interestingly, with R5 alone, encapsulation of DGK at a level of more than 50% was achieved (Table 1). This observation points to the fact that an efficient coprecipitation occurs, in contrast to what was previously observed for soluble proteins such as thioredoxin and GFP.^15^

**Table 1:** DGK Encapsulation Efficiency of R5 Peptide and BP-1 Variants

silica-precipitating peptide	R5	BP-1	R5-BP-1	BP-1-R5
DGK encapsulation efficiency	58% ± 13%	13% ± 5%	21% ± 10%	81% ± 4%
peptide integration	29% ± 8%	n.a.^a^	34% ± 8%	25% ± 3%

a n.a.: not applicable.

**Figure 5:**
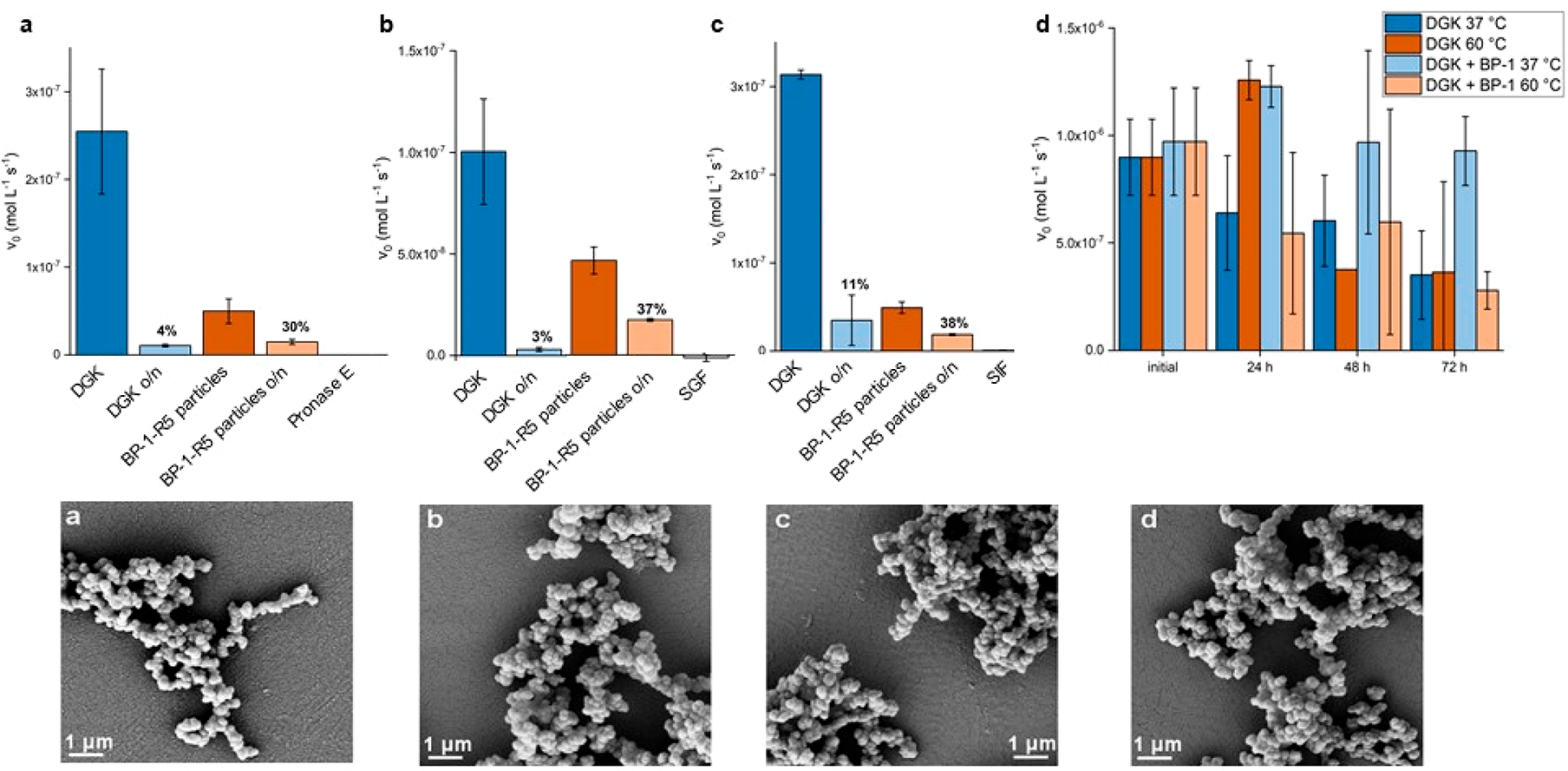
Top: Stability assays of silica-encapsulated DGK. (a) Pronase E assay. (b) Simulated gastric fluid assay. (c) Simulated intestinal fluid assay. Sample activities were measured before and after incubation overnight (o/n) as described in the Materials and Methods section. (d) Activity of DGK incubated at 37 and 60 °C for 3 days with or without 20 equiv of BP-1. Bottom: Scanning electron micrographs of DGK BP-1-R5 silica particles. (a) Treated with Pronase E. (b) Treated with SGF. (c) Treated with SIF. (d) Control. Samples: diacylglycerol kinase (DGK), silica particles from R5 and DGK coprecipitation (R5 part.), silica particles from BP-1-R5 and DGK (BP-1-R5 part.), Pronase E negative control (Pron. E), simulated gastric fluid negative control (SGF), and simulated intestinal fluid negative control (SIF).

To evaluate whether DGK solubilized by the BP-1 peptide alone, in the absence of a silica shell, is more stable than DGK in detergent solution, the phosphorylation activity of the enzyme was measured after incubation at 37 and 60 °C for up to 72 h in the presence and absence of 20 equiv of BP-1 (Figure 5a).

For DGK without BP-1, a decrease in activity of ∼50% was observed at 37 °C over 72 h. The addition of BP-1 has a protective effect and completely prevents the loss in DGK activity over 72 h within the experimental error (Figure 5a). This behavior has been reported before and is based on the shielding effect of the lipidated peptide BP-1. The initial results for the incubation at 60 °C were unexpected, as the activity for the untreated sample (which was used as purified with *n*-dodecyl-β-d-maltoside (DDM)) increased after 24 h of incubation. After 72 h, however, the activity decreased to nearly the same value as found at 37 °C. At 60 °C, the addition of BP-1 had no stabilizing effect on DGK, most likely due to losing its stabilizing structure at the elevated temperature, e.g., a cylindrical β-barrel as reported for other β-sheet forming peptides.^11,40^

For DGK encapsulated in silica particles, a different behavior was observed, and to evaluate a potential protective effect of DGK encapsulation in silica after coencapsulation with R5 or stabilization of the enzyme with BP-1-R5, enzyme activity was measured by comparing free or encapsulated DGK after incubation with Pronase E, simulated gastric fluid (SGF), or simulated intestinal fluid (SIF). The latter conditions mimic the harsh environments in the stomach (with the protease pepsin at pH 1.2) and in the intestine (with a mix of digestive enzymes containing amylase, lipases, and proteases at pH 6.8), respectively. The incubation with Pronase E abolishes 96% of the activity of the free enzyme, whereas the encapsulated enzyme retains 30% of its activity (Figure 5a). Thus, even though the basal activity of the encapsulated enzyme is lower, most likely due to limited diffusion of the rather large and hydrophobic substrate into the silica matrix, a protective effect of silica encapsulation against protease degradation is observed here. More detrimental conditions were tested as well by exposing DGK preparations to simulated gastric fluid (Figure 5b) and simulated intestinal fluid (Figure 5c). In both cases, the relative stabilization using the BP-1-R5 peptide for silica encapsulation was higher than that of coencapsulation with R5 only. In order to ensure that silica particles remained unchanged after these treatments, scanning electron microscopy images were collected. No differences to particles prior to exposure to Pronase E, SGF, and SIF or after storage under physiological conditions were observed (Figure 5a–d).

## Conclusions

We set out to generate integral membrane proteins in silica, a goal hardly achievable with commonly used silica precipitation processes.^41^ In a first step, we successfully generated silica-stabilized nanodiscs by designing novel silaffin peptide (R5)-MSP fusion constructs that expressed with high yields in *E. coli*. The best-performing construct with N- and C-terminal fusions of the R5 peptide (R5-MSP-R5) was purified, and incubation with lipids led to the formation of lipid nanodiscs with similar properties as found for MSP-only-based nanodiscs (Figure 2). Subsequent exposure of R5-MSP-R5 containing nanodiscs to freshly generated silicic acid under physiological conditions gave rise to silica-covered stacks of nanodiscs (Figure 3). Such defined membrane structures are a unique nanomaterial that can serve as a novel device for long-term storage and application of membrane proteins for sensors or as catalysts in biotechnology and medicine, or they might find applications in optical nanodevices or nanomotors.^42^ We can also envisage their use as delivery tools in cell biology and medical applications, as silica and lipids in such assemblies are typically considered nontoxic.^43^ To this end, we will also study the further functionalization of these silica nanodiscs to equip them with additional properties.^44^ Generating silica-stabilized membrane proteins or cell-based reactors has been attempted for several decades by coating cells with silica either by the direct addition of silica particles^45^ or by employing charged polymers in combination with silicic acid.^46^ Our approach, however, provides well-defined membrane discs, and their diameter can be adapted by using different MSP constructs as well as different lipid compositions.^7^ Unfortunately, we did not succeed in loading these nanodiscs with functional DGK, the model IMP selected here, since it is a rare integral membrane enzyme for which a functional assay is easily available.^25^ We will explore this route further by including other IMPs for which the successful incorporation into nanodiscs has been described.^7^ However, we also expect that the individual properties of different types of membrane proteins make it quite challenging to directly transfer such encapsulation conditions from one to another, and therefore, viable alternatives are needed.

Therefore, our second approach was based on modified β-strand peptides and is partially orthogonal to the one described above as it omits the need for lipids that can complicate the application of such stabilized membrane protein preparations. By extending the BP-1 peptide^11^ with the silaffin R5 sequence, we were able to generate a synthetic construct stabilizing DGK and inducing subsequent silica particle formation. Limited solubility prevented the use of R5′ fusion constructs with BP-1, since the shorter KRRIL extension did not convey sufficient solubility in aqueous buffers. Future explorations of this approach could utilize the R5 or R5′ motif in combination with other peptide-based, detergent-free membrane protein stabilizers such as the beltide^9^ or peptidisc^10^ systems. The R5-BP-1- and BP-1-R5-based spherical silica particles containing DGK (Figure 4) successfully proved DGK activity by converting diacylglycerol to phosphatidic acid, although with reduced activity, most likely due to the limited access of the diacylglycerol substrate to DGK inside the particles (Figure 5). The latter was indirectly demonstrated by the increasing DGK activity after preincubation of particles with the hydrophobic diacylglycerol substrate. Encapsulated DGK demonstrated an increased stability toward different proteolytic media, bringing our goal of using stabilized IMPs under stress conditions, and therefore for a variety of applications such as their use in biosensing, as a carrier for otherwise water-insoluble enzymes in biotechnology, in drug screening, or energy storage, within reach. It should be noted here that additional work is necessary to ensure that this approach is transferrable to other, more complex classes of membrane proteins with well-described functionality such as rhodopsin.^47^

## Materials and Methods

### General

A Milli-Q Reference A+ water purification system by Merck GmbH (Vienna, Austria) was used to further purify water deionized with a Professional G7895 Aqua Purificator by Miele GmbH (Salzburg, Austria). Solvents were obtained from VWR International LLC (Vienna, Austria) of HPLC grade or peptide synthesis grade. Resins were obtained from Iris Biotech (Marktredwitz, Germany) and Novabiochem by Merck GmbH (Vienna, Austria). Fmoc-protected amino acids were obtained from Alfa Aesar by Thermo Fisher Scientific GmbH (Vienna, Austria). Lipids were obtained from Avanti Polar Lipids, Inc. (Alabaster, AL). *Streptomyces griseus* Pronase E was obtained from F. Hoffmann-La Roche AG (Vienna, Austria). All other chemicals were obtained from Sigma-Aldrich by Merck GmbH (Vienna, Austria) unless otherwise specified. Restriction enzymes, buffers, and markers were obtained from New England Biolabs GmbH (Frankfurt am Main, Germany). Gel extraction kits were obtained from Invitrogen by Thermo Fisher Scientific GmbH (Vienna, Austria). Plasmid extraction was performed using the GeneJET Plasmid Miniprep kit also from Thermo Fisher Scientific GmbH (Vienna, Austria). *n*-Octyl-β-d-glucoside (OG) and *n*-dodecyl-β-d-maltoside (DDM) were obtained from Gerbu Biotechnik GmbH (Heidelberg, Germany). Ingredients for media were obtained from AppliChem (Darmstadt, Germany) of microbiology grade. Spectrapor dialysis membranes were obtained from Spectrum Chemical Mfg. Corp. (New Brunswick, NJ). Spin filters were Amicon Ultra centrifugal filters by Merck GmbH (Vienna, Austria). Zeba spin desalting columns were obtained from Thermo Fisher Scientific GmbH (Vienna, Austria). Spectra were recorded in 50 mM potassium phosphate buffer (pH 7). Absorbance was measured using a Thermo Scientific NanoDrop 2000c spectrophotometer.

### Electron and Atomic Force Microscopy

Scanning electron microscopy was performed at a 5 kV acceleration voltage on a Supra 55 VP instrument by Carl Zeiss AG (Oberkochen, Germany) equipped with an EDX detector after sputter coating the samples with 5 nm of gold using an EM QSG 100 instrument by Leica Camera AG (Wetzlar, Germany). Transmission electron microscopy was performed at a 20 kV acceleration voltage on a CM200 system by Philips GmbH (Vienna, Austria) equipped with an Orius SC600 CCD camera by Gatan GmbH (Munich, Germany). Carbon film coated copper grids (200 mesh) were obtained from Plano GmbH (Marburg, Germany). SEM samples were spotted onto 13 mm Nunc Thermanox cell culture coverslips from Thermo Fisher Scientific GmbH (Vienna, Austria). Atomic force micrographs were recorded on a Park NX10 instrument by Park Systems Corp. (Suwon, South Korea) using a silicon tip with a nominal radius below 10 nm operating in tapping mode.

### Peptide Synthesis

Peptides were synthesized manually or on a Liberty blue microwave peptide synthesizer (CEM GmbH, Kamp-Lintfort, Germany) or on a Tribute peptide synthesizer (Gyros Protein Technologies, Inc., Manchester, U.K.) using Fmoc-protected amino acids. Side-chain protecting groups used were Arg(Pbf), Lys(Boc), Ser(tBu), and Tyr(tBu) on a polystyrene resin (100–200 mesh) with a Wang (*p*-alkoxybenzyl alcohol) linker^48^ preloaded with leucine for the BP-1-KRRIL and BP-1-R5 peptides or a Rink amide AM resin (100–200 mesh) for the BP-1, KRRIL-BP-1, and R5-BP-1 peptides at scales between 0.05 and 0.2 mmol. The pseudoproline dipeptide Fmoc-Gly-Ser(ΨMe,Mepro)–OH was used at suitable positions in the sequence, as described previously.^37^ In addition, the noncanonical amino acids Fmoc-(*N*-met)Leu-OH and Fmoc-(Octyl)Gly-OH were used. Amino acids were activated with HATU for *N*-methyl-l-leucine and HCTU for all other amino acids. Double couplings were performed for serine following *N*-methyl-l-leucine. N-terminal acetylation was performed with a 3:2:1 mixture of DMF, pyridine, and acetic anhydride. After cleavage from the resin, peptides were precipitated in diethyl ether. The unmodified BP-1 peptide, however, did not precipitate under these conditions. Accordingly, for this peptide, the cleavage solution was diluted 100 times with water/ACN (1:1) and lyophilized to obtain the crude product.

Purification by RP-HPLC was carried out on either a ProStar system by Varian, Inc., now Agilent Technologies (Santa Clara, CA), or an Open Architecture HPLC system by Waters S. A. S. (Saint-Quentin, France). For analytical HPLC-MS, either the Open Architecture HPLC system by Waters S. A. S. (Saint-Quentin, France) or an UltiMate 3000 HPLC system equipped with an MSQ Plus mass spectrometer by Thermo Fisher Scientific GmbH (Vienna, Austria) was used.

### Protein Synthesis and Purification

Protein synthesis and purification of MSP and MSP-R5 fusion constructs were performed as described by Sligar et al.^14^ MSP constructs were expressed in *E. coli* BL21 (DE3) Rosetta 2 transformed with the expression plasmid pET 28a containing the desired insert. Affinity chromatography was performed on an ÄKTAprime plus LC system by GE Healthcare (Vienna, Austria). HisTrap high-performance Ni-affinity columns were used, also by GE Healthcare (Vienna, Austria).

The method used for the expression and purification of DGK was modified from the one described by Lau et al.^25^ The protein was produced in *E. coli* BL21 (DE3) transformed with the plasmid pSD004. The protein was solubilized directly from the whole bacteria by resuspending the pellet in 40 mL of DGK solubilization buffer at 4 °C for 3 days. Afterward, it was cleared by centrifugation at 20 000 rpm for 30 min before purification using Ni-affinity chromatography.

### Synthesis of Silica Particles

Silica particles were precipitated from a 1 mg/mL solution of the silica-precipitating peptide/protein in 50 mM potassium phosphate buffer at pH 7. The concentration was determined by weighing the lyophilized peptide. This solution was then incubated overnight at room temperature. A silicic acid solution was generated by adding 40 μL of TMOS to 960 μL of 1 mM HCl, short vortexing, and incubation of the mixture for 4 min. A 10 μL portion of this silicic acid solution was added to a 90 μL aliquot of the peptide/protein solution. The mixture was vortexed and incubated for 30 min at room temperature. It was then centrifuged at 14 000 rpm for 5 min. After removing the supernatant, the particles were washed with water/buffer and the particles dried at reduced pressure.

### Formation of Nanodiscs

The method used to induce the formation of lipid-bilayer nanodiscs was based on the protocol by Luthra et al.^49^ 1,2-Dioleoyl-*sn*-glycero-3-phosphocholine (DOPC) was solubilized at a 50 mM concentration with the following method. 25.6 mg (32.6 μmol) of the lipid was dissolved in 2.5 mL of chloroform in a glass tube. The solvent was evaporated in an argon stream while the tube was turned at an angle. The lipid-coated glass tube was dried overnight in the desiccator. The lipid was again dissolved in 650 μL of a solution of 100 mM sodium cholate and 100 mM NaCl by repeated cycles of heating to 60 °C and sonicating for 3 min. The process was repeated until the solution was completely clear.

A 500 μL nanodisc formation solution was prepared from 70 μL of solubilized DOPC (7 mM final concentration) by adding MSP washing buffer 3 and purified protein to a final protein concentration of 50 μM (1:140 ratio of protein to lipid). This was incubated for 30 min on ice. After incubation, the mixture was transferred to a prehydrated dialysis cassette (10 kDa MWCO) and dialyzed against 200 mL of MSP washing buffer 3. The buffer was changed 2 times and the final dialysis step run overnight.

### Kinase Activity Assay

To measure the kinase activity of the DGK samples, the assay published by Lau et al. was used.^25^ The spectrometer was blanked against the DGK dialysis buffer. A 10 μL portion of the sample was then added and mixed in and the absorbance at 340 nm was monitored over 30 min every 2 s. For the nanoparticle samples, 20 μL of resuspended nanoparticles was preincubated with 10 μL of dioleylglycerol (7 mM, in DMSO) for 20 min before the mixture was added to the cuvette. Accordingly, the volume of buffer was reduced to 362 μL.

### Stability Assays

The encapsulated and free DGK enzymes were subjected to three different conditions to evaluate stabilization due to silica encapsulation.

#### Pronase E

To a 100 μL sample (1 mg/mL DGK or particles created with the same amount) were added 1 μL of a Pronase E stock solution (20 mg/mL) and 1 μL of CaCl_2_ solution (1 M). The mixture was incubated at 40 °C for 16 h. Afterward, 20 μL aliquots were made, flash frozen in liquid nitrogen, and stored on ice. A negative control was prepared from 100 μL of buffer without DGK or particles and treated in the same way. Activity tests were then performed as described above.

#### Simulated Gastric Fluid Assay

Simulated gastric fluid (SGF) was prepared according to the specifications provided by the United States Pharmacopeia (USP) and the National Formulary (NF-USP 41-NF 36). 100 μL samples were first lyophilized and then resuspended in 100 μL of SGF (0.8 g/L porcine pepsin, 34 mM NaCl, pH 1.2) and incubated for 16 h at 37 °C. Afterward, 20 μL aliquots were prepared, flash frozen in liquid nitrogen, and stored on ice. A negative control was prepared by incubating 100 μL of SGF without particles or DGK and treating it in the same way. Activity tests were then performed as described above.

#### Simulated Intestinal Fluid Assay

Simulated intestinal fluid (SIF) was prepared according to the specifications provided by the United States Pharmacopeia (USP) and the National Formulary (NF-USP 41-NF 36) by mixing 100 mg of pancreatin with 10 mL of 50 mM potassium phosphate buffer pH 6.8. The mixture was vortexed, sonicated for 15 min at 25 °C, and then centrifuged and filtered through a 20 μm syringe filter. Samples were first lyophilized and then resuspended in 100 μL of SIF and incubated for 16 h at 37 °C. Again, 20 μL aliquots were prepared, flash frozen in liquid nitrogen, and stored on ice. A negative control was prepared by incubating 100 μL of SIF without particles or DGK and treating it in the same way. Activity tests were then performed as described above.

## Supporting Information Available

Detailed information on cloning procedures, peptide synthesis, and characterization as well as on AFM, EM, and EDXA measurements (PDF)

## Abbreviations

AFM: atomic force microscopy
DGK: diacylglycerol kinase
HPLC: high-performance liquid chromatography
IMP: integral membrane protein
MSP: membrane scaffold protein
NF: National Formulary
SEC: size-exclusion chromatography
SEM: scanning electron microscopy
SGF: simulated gastric fluid
SIF: simulated intestinal fluid
SMALP: styrene maleic acid lipid particles
SPPS: solid-phase peptide synthesis
TEM: transmission electron microscopy
TFA: trifluoroacetic acid
TMOS: tetramethyl orthosilicate
USP: United States Pharmacopeia
