# Supplementary material for: Biomimetic Silica Encapsulation of Lipid Nanodiscs and β-Sheet-Stabilized Diacylglycerol Kinase
Source: Bioconjug Chem. 2021 Jul 21;32(8):1742–52. doi: 10.1021/acs.bioconjchem.1c00260 (PMC8382255; doi:10.1021/acs.bioconjchem.1c00260)
Supplement: Supplementary file 1 — bc1c00260_si_001.pdf [file bc1c00260_si_001.pdf]

# Supporting information

# Biomimetic Silica Encapsulation of Lipid Nanodiscs and β-Sheet Stabilized Diacylglycerol Kinase

*Friedrich Bialas and Christian F. W. Becker**

Institute of Biological Chemistry, Faculty of Chemistry, University of Vienna, Währinger Straße 38, 1090 Vienna, Austria

*Corresponding author – Email: christian.becker@univie.ac.at

## Buffers and expression media

**MSP Lysis buffer**

- 20 mM $NaH_2PO_4$, pH 7.4
- 1 mM PMSF
- 1% Triton-X100

**MSP Equilibration buffer**

- 20 mM $NaH_2PO_4$, pH 7.4
- 1% Triton-X100

**MSP Washing buffer 1**

- 40 mM Tris-HCl, pH 8.0
- 0.3 M NaCl
- 1% Triton

**MSP Washing buffer 2**

- 40 mM Tris-HCl, pH 8.0
- 0.3 M NaCl
- 50 mM sodium cholate

**MSP Washing buffer 3**

- 40 mM Tris-HCl, pH 8.0

· 0.3 M NaCl

**MSP Elution buffer**

· 40 mM Tris-HCl, pH 8.0
· 0.3 M NaCl
· 0.3 M imidazole

**DGK Solubilization buffer**

· 50 mM sodium phosphate, pH 7.5
· 300 mM NaCl
· 1 mM phenylmethylsulfonyl fluoride (PMSF)
· 3% n-octyl-β-d-glucoside (OG)

**DGK Equilibration buffer**

· 50 mM sodium phosphate, pH 7.5
· 300 mM NaCl

**DGK Washing buffer**

· 50 mM sodium phosphate, pH 8
· 300 mM NaCl
· 250 mM imidazole

**DGK elution buffer**

· 50 mM sodium phosphate-HCl, pH 8
· 300 mM NaCl
· 250 mM imidazole
· 0.5% n-dodecyl β-D-maltoside (DDM)

**DGK dialysis buffer**

· 50 mM sodium phosphate, pH 8
· 300 mM NaCl

**TEM negative staining solution**

· 1% phosphotungstic acid-NaOH, pH 7.2

**CD buffer**

· 100 mM Tris-HCl, pH 7.35
· 40 mM NaCl

## R5-MSP-R5 cloning

The MSP1E3D1-containing pET28a plasmid was sequenced with the T7 terminator and T7 promoter primers and the sequence searched for unique restriction sites. Four sites (NcoI, BmgBI, EcoRI and NotI) were identified in the insert (see fig. S1a). The translated insert sequence is shown in figure S1b with the protein of interest marked in black. A test digest was then performed on the plasmid with these enzymes to confirm the location of the recognition sites. The resulting gel is shown in figure S1c. As expected, the plasmid is linearized when digested with any of the restriction endonucleases, leading to the appearance of a single band around 5,000 bp. When performing the dual digest with NcoI and EcoRI, a 116 bp fragment is cut out; the dual digest with BmgBI and NotI leads to the appearance of a fragment with 212 bp slightly higher on the gel. Accordingly, these restriction sites were used to introduce

inserts with the desired N- and C-terminal modifications contained in the sequence (compare fig. S2). Table 1 shows the resulting properties of R5-MSP-R5.

**a**

AGAAATAATTTTGTTTAACTTTAAGAAGGAGATATA C|CATGG GTCATCATCATCATCATCACATTGAGGGACGTCTGAA
GCTGTTGGACAATTGGGACTCTGTTACGTCTACCTTCAGTAAACTTCGCGAACAACTGGGCCCCGTGACGCAG G|AATTC
TGGGACAACCTGGAAAAAGAAACCGAGGGACTGCGTCAGGAAATGTCCAAAGATTTAGAAGAGGTGAAGGCCAAGGTTCAGC
CATATCTCGATGACTTTCAGAAAAAATGGCAGGAAGAGATGGAATTATATCGTCAAAAGGTGGAACCGCTGCGTGCGGAACT
GCAAGAGGGGGCACGCCAAAAACTCCATGAGCTCCAAGAGAAGCTCAGCCCATTAGGCGAAGAAATGCGCGATCGCGCCCGT
GCACATGTTGATGCACTCCGGACTCATTTGGCGCCATATCTCGATGACTTTCAGAAAAAATGGCAGGAAGAGATGGAATTAT
ATCGTCAAAAGGTGGAACCGCTGCGTGCGGAACTGCAAGAGGGGGCACGCCAAAAACTCCATGAGCTCCAAGAGAAGCTCAG
CCCATTAGGCGAAGAAATGCGCGATCGCGCCCGTGCACATGTTGATGCACTCCGGACTCATTTGGCGCCGTATTCGGATGAA
CTTCGCCAGCGTTTGGCCG
CAC|GTC TCGAGGCGCTGAAAGAAAACGGGGGTGCCCGCTTGGCTGAATACCACGCGAAAGCGACAGAACACCTGAGCACC
TTGAGCGAAAAAGCGAAACCGGCGCTGGAAGATCTACGCCAGGGCTTATTGCCTGTTCTTGAGAGCTTTAAAGTCAGTTTTC
TGTCAGCTCTGGAAGAATATACTAAAAAGCTGAATACCCAGTAATAAGCTT GC|GGCCGC ACTCGAGCACCACCACCACC
ACCACTGAGATCCGGCTGCTAACAAAGCCCGAAAGAG

**b**

K-FCLTLRRRYTMGHHHHHHIEGRLKLLDNWDSVTSTFSKLREQLGPVTQEFWDNLEKETEGLRQEMSKDLEEVKAKVQPYL
DDFQKKWQEEMELYRQKVEPLRAELQEGARQKLHELQEKLSPLGEEMRDRARAHVDALRTHLAPYLDDFQKKWQEEMELYRQ
KVEPLRAELQEGARQKLHELQEKLSPLGEEMRDRARAHVDALRTHLAPYSDELRQRLAARLEALKENGGARLAEYHAKATEH
LSTLSEKAKPALEDLRQGLLPVLESFKVSFLSALEEYTKKLNTQ--ACGRTRAPPPPPLRSGC-QSPKE

**c**

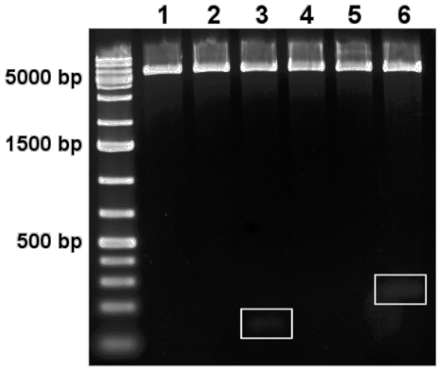


**Figure S1. MSP1E3D1 sequence. a**, sequencing results with recognition sites for NcoI (blue), EcoRI (red), BmgBI (yellow), and NotI (green). **b**, derived amino acid sequence with the translated protein marked in black. **c**, test digest of the MSP1E3D1-pET28a plasmid. Lanes: NcoI (1), EcoRI (2), NcoI + EcoRI (3), BmgBI (4), NotI (5), BmgBI + NotI (6). The cut out fragments are marked with boxes.

**a**

C|CATGG GTCATCATCATCATCATCATGAAAACCTGTATTTTCAGGGCAGCACCTTTAGCAAACTGCGCGAACAGCTGGGC
CCGGTGACCCAG G|AATTC

MGHHHHHHENLYFQGSTFSKLREQLGPVTQEF

**b**

C|CATGG GTCATCATCATCATCATCATGAAAACCTGTATTTTCAGGGCAGCAGCAAAAAAAGCGGCAGCTATAGCGGCAGC
AAAGGCAGCAAACGCCGCATTCTGAGCACCTTTAGCAAACTGCGCGAACAGCTGGGCCCGGTGACCCAG G|AATTC

MGHHHHHHENLYFQGSSKKSGSYSGSKGSKRRILTFSKLREQLGPVTQEF

**c**

CAC|GTC TCGAGGCGCTGAAAGAAAACGGCGGCGCGCGCCTGGCGGAATATCATGCGAAAGCGACCGAACATCTGAGCACC
CTGAGCGAAAAAGCGAAACCGGCGCTGGAAGATCTGCGCCAGGGCCTGCTGCCGGTGCTGGAAAGCTTTAAAGTGAGCTTTC
TGAGCGCGCTGGAAGAATATACCAAAAAACTGAACACCCAGAGCAGCAAAAAAAGCGGCAGCTATAGCGGCAGCAAAGGCAG
CAAACGCCGCATTCTGTAATAAGCTTGC|GGCCGC

RLEALKENGGARLAEYHAKATEHLSTLSEKAKPALEDLRQGLLPVLESFKVSFLSALEEYTKKLNTQSSKKSGSYSGSKGSK
RRIL--ACGR

**Figure S2.** Insert design. **a**, nucleotide sequence of the N-terminal TEV-site insert with the recognition sites for NcoI (blue) and EcoRI (red) and translated amino acid sequence with the TEV protease cleavage site marked in orange. **b**, nucleotide sequence of the N-terminal R5 insert with the recognition sites for NcoI (blue) and EcoRI (red) and translated amino acid sequence with the R5 peptide sequence marked in orange. **c**, nucleotide sequence of the C-terminal insert with the recognition sites for BmgBI (yellow) and NotI (green) and translated amino acid sequence with the R5 peptide sequence marked in orange.

**Table S1. Properties of the R5-MSP-R5** before and after the cleavage of the His-tag at the TEV protease recognition site.

| **protein** | **number of aa** | **MW** | **pI** | **$\varepsilon_{280nm}$** |
|---|---|---|---|---|
| $His_6$-TEV-R5-MSP-R5 | 307 | 35,647 Da | 8.91 | 31,400 $M^{-1}$ $cm^{-1}$ |
| R5-MSP-R5 | 294 | 33,972 Da | 9.06 | 29,910 $M^{-1}$ $cm^{-1}$ |

## Expression of MSP constructs

The constructs were expressed and purified according to Bayburt *et al.*[1] and yielded the analysis results shown in figure S3. CD spectra of both proteins were collected as shown in figure S4.

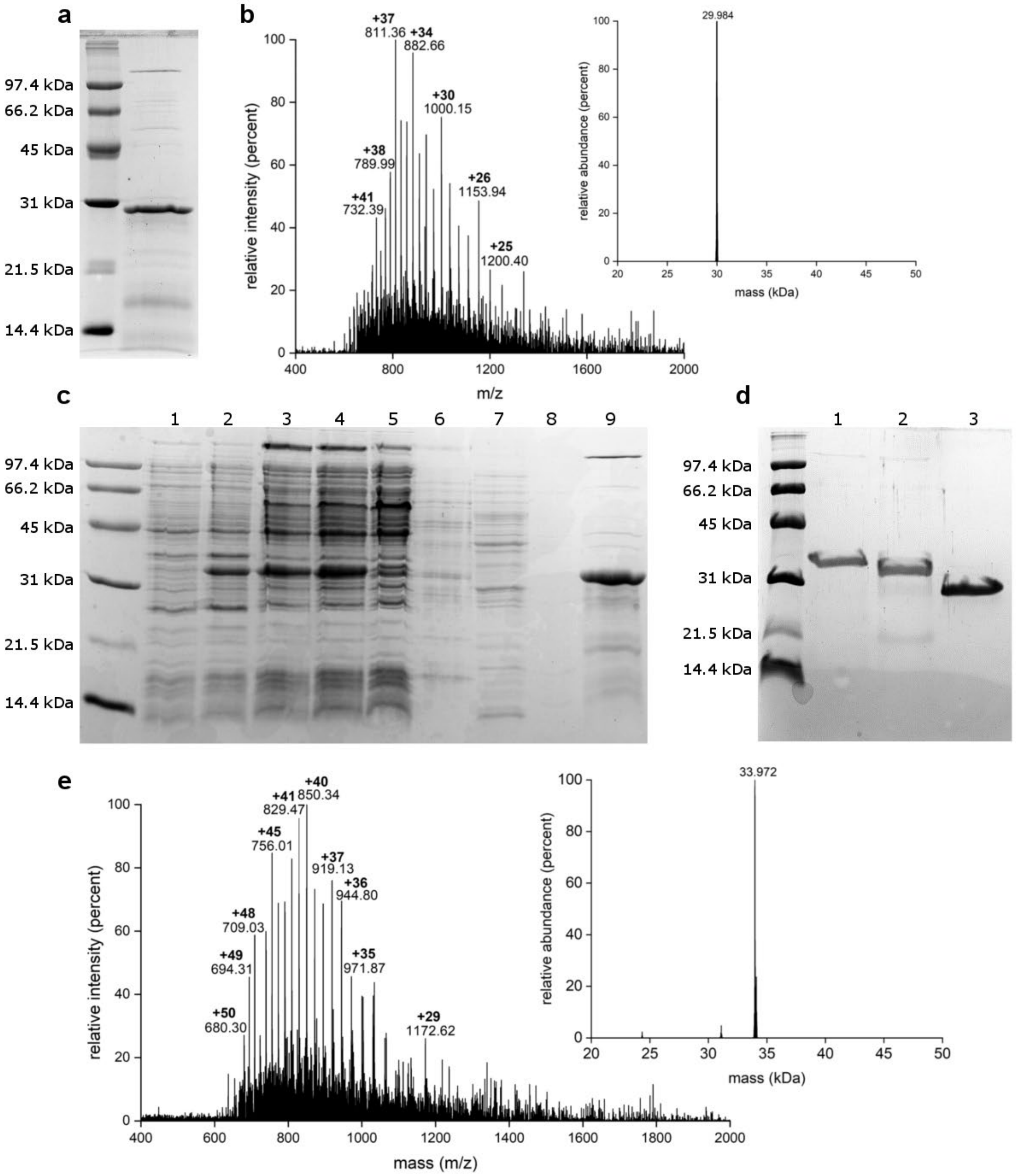


**Figure S3. Analysis of MSP proteins.** SDS-PAGE (**a**) and LC-MS mass spectrum and deconvoluted mass spectrum (insert) of the MSP protein (**b**), calc. mass 29,982 Da. SDS-PAGE of the R5-MSP-R5 protein purification (**c**), Samples taken: before induction (1), after 3 h expression (2), lysate (3), supernatant (4), flow-through (5), wash 1-3 (6-8), eluent (9) and TEV protease digest (**d**), before (1) and after (2) treatment. LC-MS Mass spectrum and deconvoluted mass spectrum (insert) of the cleaved R5-MSP-R5 protein (**e**), calc. mass 33,972 Da.

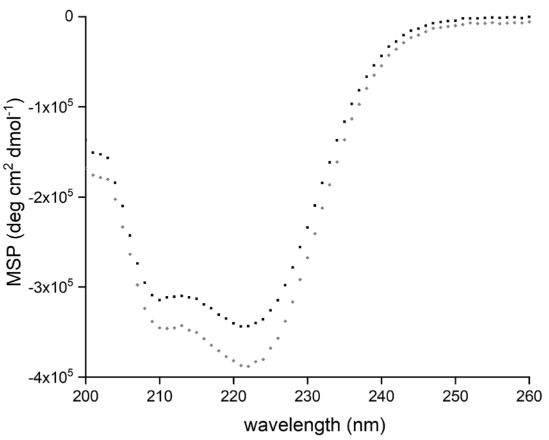


**Figure S4. CD spectra** of the MSP (black squares) and R5-MSP-R5 (grey diamonds) proteins at 10 µM concentration. Both variants show minima at 210 nm and 222 nm, indicating an alpha helical conformation.

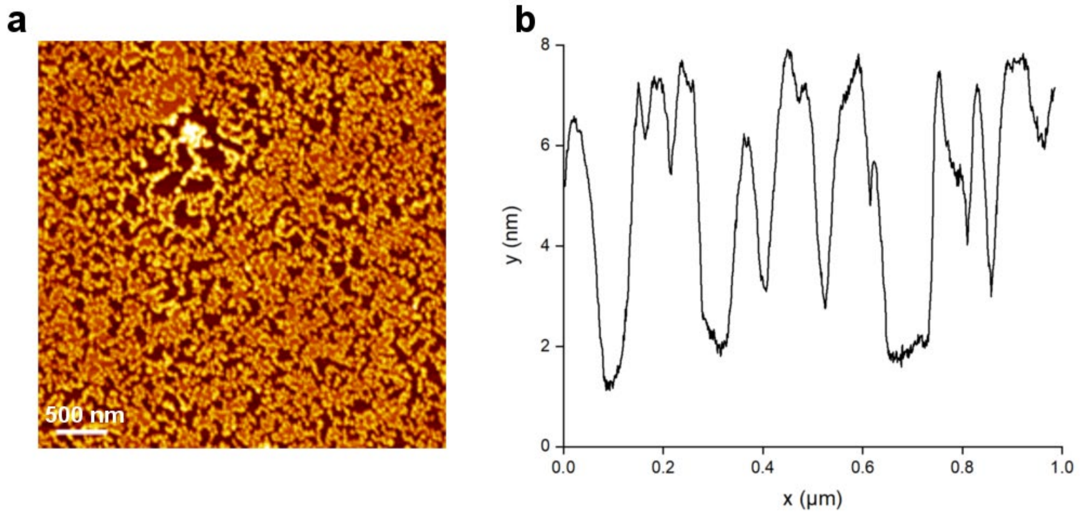


**Figure S5 | AFM analysis of R5-MSP-R5 nanodiscs. a**, Individual nanodiscs are visible as dots on a mica surface. **b**, A line plot reveals areas covered by nanodiscs are approximately 6 nm higher than free areas. The plot was generated using the Gwyddion software.

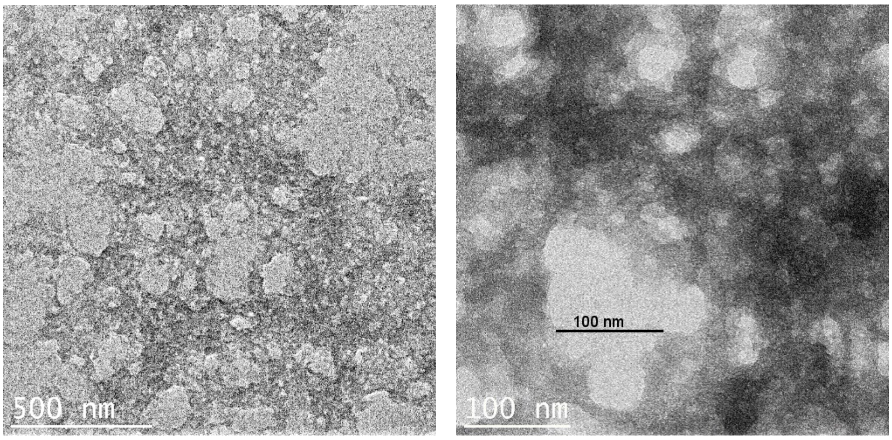


**Figure S6. Transmission electron micrographs** of silica particles precipitated with the R5-MSP-R5 protein at increasing magnification.

## Expression and purification of diacylglycerol kinase

Diacylglycerol kinase (DGK) was expressed in *E. coli* BL21 (DE3) from the plasmid psD004 and purified according to the protocol used by Lau *et al.*[2]. The detergents n-octyl-β-D-glucoside (OG) and n-dodecyl-β-D-maltoside (DDM) were used to solubilize and elute the protein, respectively. Figure S5 shows the sequencing and analysis data.

**a**

```
AAAATTAAAGAGGTATATATTAATGTATCGATTAAATAAGGAGGAATAAACCATGGGGCATCATCATCATCATCATGAGCTC
GCTAACAACACCACCGGTTTCACCCGTATCATCAAAGCGGCCGGTTACAGCTGGAAAGGCCTGCGTGCTGCTTGGATCAACG
AAGCTGCATTCCGTCAGGAAGGTGTTGCTGTTCTGCTGGCTGTTGTTATCGCTTGCTGGCTGGACGTCGACGCTATCACCCG
TGTTCTGCTGATCTCGAGCGTTATGCTGGTTATGATCGTTGAAATCCTGAATTCCGCTATCGAAGCTGTTGTTGACCGTATC
GGATCCGAATACCACGAACTGAGCGGCCGCGCTAAAGACATGGGTTCTGCAGCTGTTCTGATCGCTATCATCGTTGCTGTTA
TCACCTGGTGCATCCTGCTGTGGTCCCACTTCGGTTAAGCTTGGCTGTTTTGGCGGATGAGAGAAGATTTTCAGCCTGATAC
AGATTAAATCAGAACGCAGAAGCGGTCTGATAAAACAGAATTTGCCTGGCGGCAGTAGCGCGGTGGTCCCACCTGACCCCAT
GCCGAACTCAGAAGTGAAACGCCGTAGCGCCGATGGTAGTGTGGGGTCTCCCCATGCGAGAGTAGGGAACTGCCAGGCATCA
AATAAAACGAAAGGCTCAGTCGAAAGACTGGGCCTTTCGTTTTATCTGTTGTTTGTCGGTGAACGCTCTCCTGAGTAGGACA
AATCCGCCGGGAGCGGATTTGAACGTTGCGAAGCAACGGCCCGGAAGGGTGGCGGGCAGGGACGCCCGCCATAAACTGCCAG
GCATCAAATTAAGCAGAAGGCCATCCTGACGGATGGCCTTTTTGCGTTTCTACAAACTCTTTTGTTTATTTTTCTAAATACA
TTCAAATATGTATCCGCTCATGAGACAATAA
```

```
KLKRYILMYRLNKEE-TMGHHHHHHELANNTTGFTRIIKAAGYSWKGLRAAWINEAAFRQEGVAVLLAVVIACWLDVDAITR
VLLISSVMLVMIVEILNSAIEAVVDRIGSEYHELSGRAKDMGSAAVLIAIIVAVITWCILLWSHFG-AWLFWRMREDFQPDT
D-IRTQKRSDKTEFAWRQ-RGGPT-PHAELRSETP-RRW-CGVSPCESRELPGIK-NERLSRKTGPFVLSVVCR-TLS-VGQ
IRRERI-TLRSNGPEGWRAGTPAINCQASN-AEGHPDGWPFCVSTNSFVYFSKYIQICIRS-DN
```

(130 aa, 14233.83 Da, pI 6.46, $\varepsilon_{280nm}$ = 30,480 $M^{-1}$ $cm^{-1}$)

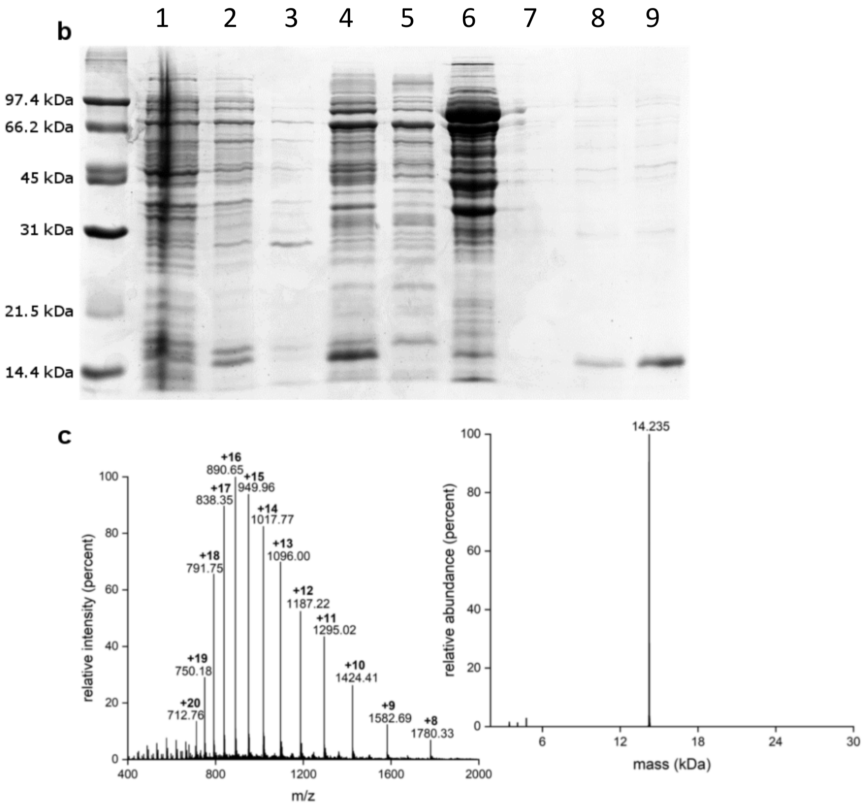


**Figure S7. Expression and purification of DGK. a**, result of nucleotide sequence and translated amino acid sequence. The protein of interest is shown in black and its properties are indicated below. **b**, expression gel. Lanes: MWM, before induction (1), overnight expression after induction (2), pellet (3), supernatant (4), flow-through (5), eluted fractions from Ni-NTA (6 - 9). **c**, mass spectrum and deconvoluted mass spectrum (insert) from LC-MS of the purified protein.

**Loading of nanodiscs with DGK**
We attempted to embed diacylglycerol kinase in the stabilized nanodiscs. Accordingly, the nanodisc formation procedure was repeated in presence of DGK. The following mixture was prepared, corresponding to 31 nmol (1 eq.) R5-MSP-R5, 4,0 µmol (131 eq.) DOPC, and 46 nmol (1.5 eq.) of DGK:

· 500 µl R5-MSP-R5 (2.095 mg/ml)

· 80,7 µl DOPC solution (50 mM DOPC, 100 mM NaCl, 100 mM sodium cholate)

· 310 µl DGK 3.2 (2.121 mg/ml)

It was incubated and dialyzed as before. Purification of DGK-loaded nanodiscs, using a Ni-NTA column (1 mL) was carried out. The sample was loaded onto the column and a gradient from 0 – 100% MSP Elution buffer in MSP Washing buffer 3 was used to elute the nanodiscs. However, an elution peak was already seen within the flow-through, indicating that the nanodiscs did not bind to the Ni-NTA column. Subsequent SDS-PAGE analysis of the flow-through and the eluent did not show any band corresponding to DGK (Figure S8).

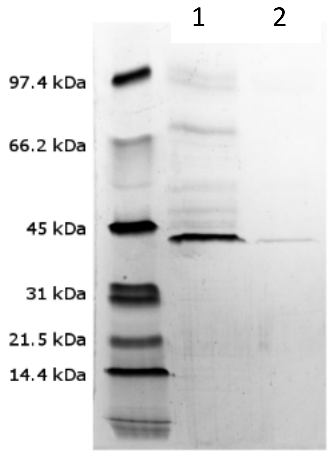


**Figure S8. SDS-PAGE** of the loading attempt of nanodiscs with DGK after Ni-NTA column purification. MWM, flow-through (1), eluate (2).

Thus, we concluded that the DGK did not integrate in the nanodiscs under these conditions and a different strategy using β-sheet peptides was used to embed the enzyme.

## Synthesis of BP-1 peptide variants

The BP-1 peptides were synthesized based on the method published by Tao *et al.*[3] and analysis yielded the results shown in figure S9. Yields are summarized in table S2. CD spectra of the peptides are shown in figure S10. Silica precipitation was performed with only the peptides (figure S11) and the encapsulation efficiency for DGK was tested (figure S12). Figure S13 shows that there was no significant degradation of the particles occurring during the Pronase E and SIF assays.

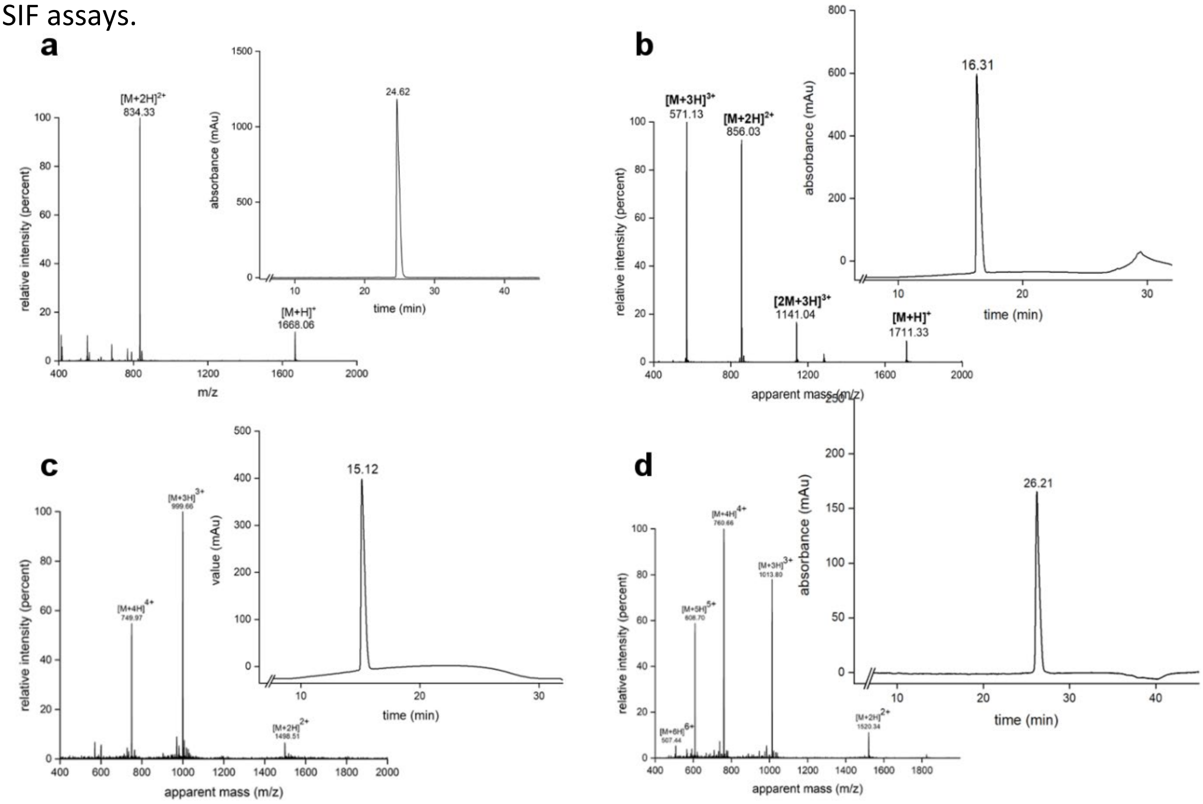


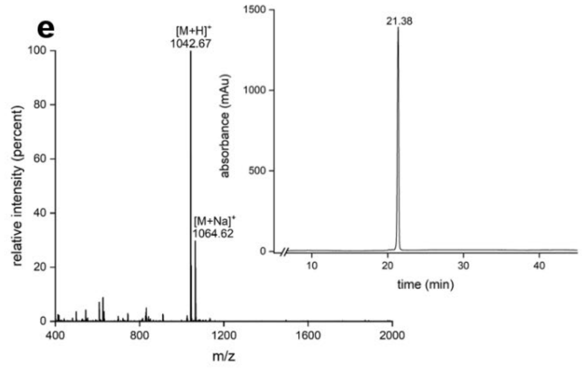


**Figure S9. Final analysis** of the peptides R5'-BP-1 (**a**), BP-1-R5' (**b**), R5-BP-1 (**c**), BP-1-R5 (**d**), and BP-1 (**e**). The mass spectrum is shown on the left, the insert shows the HPLC chromatogram (injection peaks were cut off).

**Table S2. Peptide** constructs and synthesis yields.

| peptide | aa sequence | synthesized amount (mg) | yield (%) |
|---|---|---|---|
| R5'-BP-1 | H-KRRIL-(octyl)G-SLS-(Nmet)L-D-(octyl)G-D-$NH_2$ | 74 | 88 |
| BP-1-R5' | Ac-(octyl)G-SLS-(Nmet)L-D-(octyl)G-D-KRRIL-OH | 10 | 44 |
| R5-BP-1 | H-SSKKSGSYSGSKGSKRRIL-(octyl)G-SLS-(Nmet)L-D-(octyl)G-D-$NH_2$ | 29 | 19 |
| BP-1-R5 | Ac-(octyl)G-SLS-(Nmet)L-D-(octyl)G-D-SSKKSGSYSGSKGSKRRIL-OH | 8 | 5 |

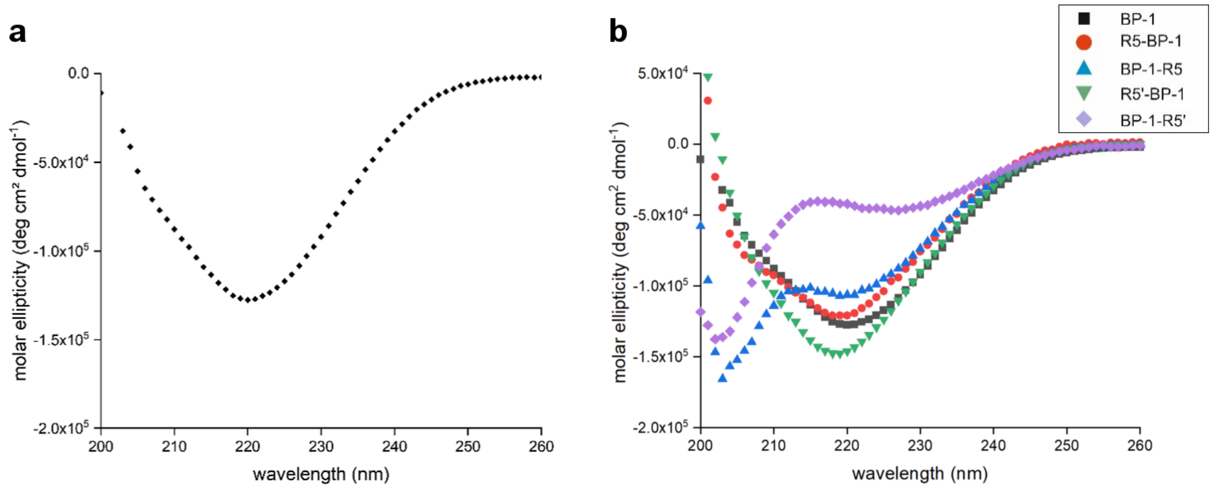


**Figure S10. CD spectra** of the BP-1 peptide alone (**a**) and in comparison with all modified BP-1 variants (**b**) at 0.5 mg/ml.

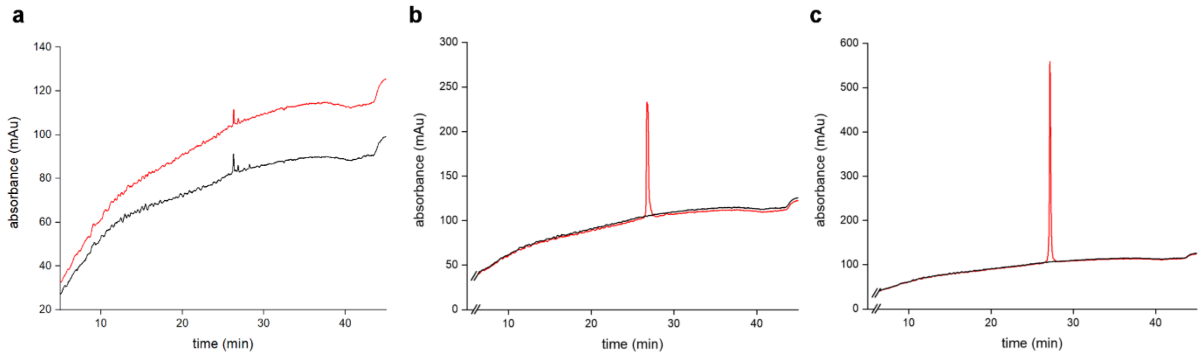


**Figure S11. HPLC chromatograms** of precipitation mixtures before the addition of silicic acid (grey curve) and of the supernatant after precipitation (black curve) with the peptides R5'-BP-1 (**a**), R5-BP-1 (**b**), and BP-1-R5 (**c**). The injection peaks were cut-off. For R5'-BP-1 (**a**) almost no signal is visible as this peptide is insoluble in the phosphate buffer required for silica precipitation.

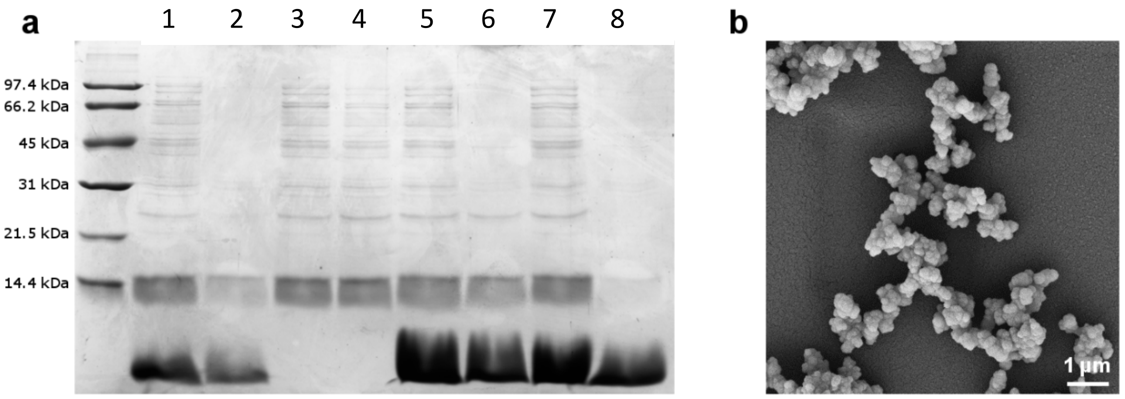


**Figure S12. DGK encapsulation efficiency. a,** SDS-PAGE: the left lane was filled with a sample from the precipitation mixture, the right lane with a sample of the supernatant after precipitation and centrifugation. DGK was precipitated with: R5 peptide (1,2), R5'-BP-1 (3,4), R5-BP-1 (5,6), and BP-1-R5 (7,8). **b**, SEM of the particles obtained by co-precipitating a mixture of the R5 peptide and the DGK protein.

## EDX Spectra

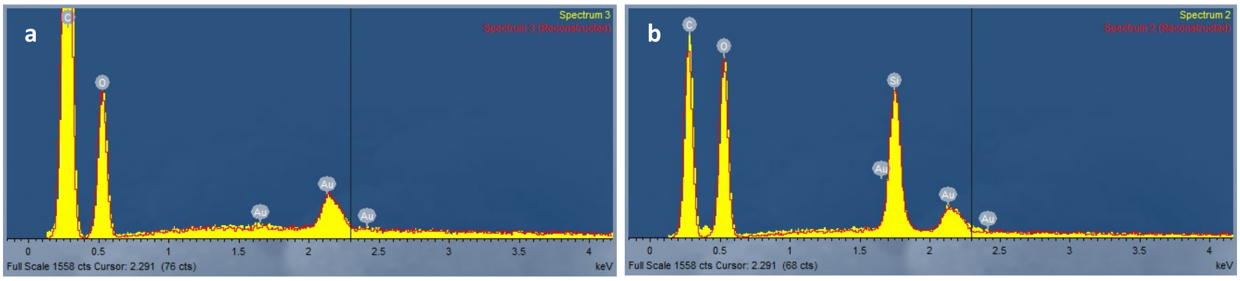


**Figure S13. EDX spectra** of R5'-BP-1 encapsulated DGK. Spectrum **a** was taken from the outer part of the cover slide (negative control). Spectrum **b** was taken from the particle sample itself. The additional peak in spectrum **b** is caused by the silicon present in the silica particles. Carbon and oxygen are from the plastic slide the samples were deposited on. The samples were sputtered with a 5 nm coating of gold, resulting in the remaining peak.

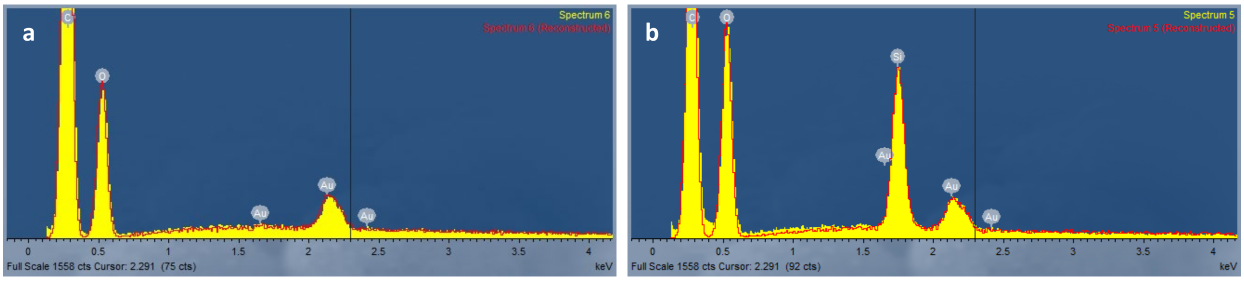


**Figure S14. EDX spectra** of R5-BP-1 encapsulated DGK. Spectrum **a** was taken from the outer part of the cover slide (negative control). Spectrum **b** was taken from the particle sample itself.

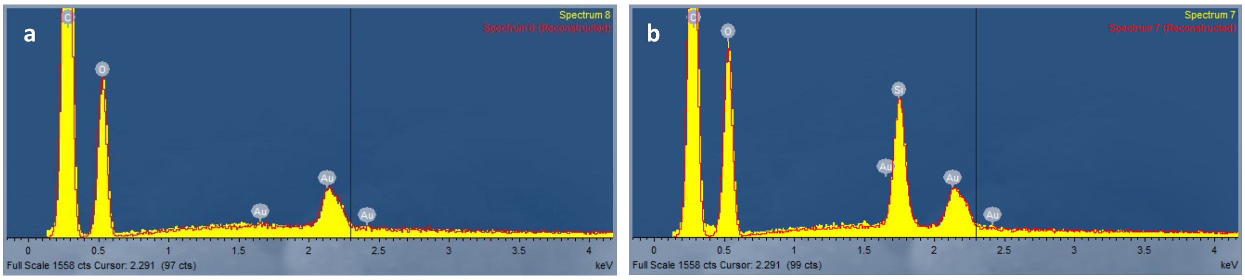


**Figure S15. EDX spectra** of BP-1-R5 encapsulated DGK. Spectrum **a** was taken from the outer part of the cover slide (negative control). Spectrum **b** was taken from the particle sample itself.

## References


(1) Bayburt, T. H., Grinkova, Y. V., and Sligar, S. G. (2002) Self-Assembly of Discoidal Phospholipid Bilayer Nanoparticles with Membrane Scaffold Proteins. *NanoLetters 2*, 853-856.

(2) Lau, F. W., and Bowie, J. U. (1997) A Method for Assessing the Stability of a Membrane Protein. *Biochemistry 36*, 5884-5892.

(3) Tao, H., Lee, S. C., Moeller, A., Roy, R. S., Siu, F. Y., Zimmermann, J., Stevens, R. C., Potter, C. S., Carragher, B., and Zhang, Q. (2013) Engineered nanostructured beta-sheet peptides protect membrane proteins. *Nat. Methods 10*, 759-761.
